# Implementation of supplemental physiotherapy following hip fracture surgery: a protocol for the process evaluation of a randomised controlled trial

**DOI:** 10.1186/s13063-024-08143-4

**Published:** 2024-05-24

**Authors:** Eleanor Raper, Lara A. Kimmel, Angela T. Burge, Ian A. Harris, Ilana N. Ackerman, Richard S. Page, Justine M. Naylor, Graham Hepworth, Belinda Gabbe, Christina L. Ekegren, Anthony Harris, Maame Esi Woode, Anne E. Holland

**Affiliations:** 1 Physiotherapy Department, Alfred Health, Melbourne, Australia; 2 Central Clinical School, Monash University, Melbourne, VIC Australia; 3 School of Public Health and Preventive Medicine, Monash University, Melbourne, Australia; 4 Institute for Breathing and Sleep, Melbourne, VIC Australia; 5 Whitlam Orthopaedic Research Centre, Ingham Institute for Applied Medical Research, Sydney, Australia; 6 School of Clinical Medicine, UNSW Medicine & Health, UNSW Sydney, Sydney, Australia; 7 Barwon Centre for Orthopaedic Research and Education, IMPACT, School of Medicine, Deakin University, Geelong, VIC Australia; 8 John of God Geelong Hospital, Geelong, VIC Australia; 9 Department of Orthopaedics, University Hospital Geelong, Barwon Health, Geelong, VIC Australia; 10 Statistical Consulting Centre, The University of Melbourne, Melbourne, VIC Australia; 11 Rehabilitation, Ageing and Independent Living (RAIL) Research Centre, School of Primary and Allied Health Care, Monash University, Melbourne, Australia; 12 Centre for Health Economics, Monash University, Melbourne, Australia

**Keywords:** Process evaluation, Implementation, Physical rehabilitation, Hip fracture, Hospitalisation

## Abstract

**Background:**

Patient outcomes following low-trauma hip fracture are suboptimal resulting in increased healthcare costs and poor functional outcomes at 1 year. Providing early and intensive in-hospital physiotherapy could help improve patient outcomes and reduce costs following hip fracture surgery. The HIP fracture Supplemental Therapy to Enhance Recovery (HIPSTER) trial will compare usual care physiotherapy to intensive in-hospital physiotherapy for patients following hip fracture surgery. The complex environments in which the intervention is implemented present unique contextual challenges that may impact intervention effectiveness. This study aims to complete a process evaluation to identify barriers and facilitators to implementation and explore the patient, carer and clinician experience of intensive therapy following hip fracture surgery.

**Methods and analysis:**

The process evaluation is embedded within a two-arm randomised, controlled, assessor-blinded trial recruiting 620 participants from eight Australian hospitals who have had surgery for a hip fracture sustained via a low-trauma injury. A theory-based mixed method process evaluation will be completed in tandem with the HIPSTER trial. Patient and carer semi-structured interviews will be completed at 6 weeks following hip fracture surgery. The clinician experience will be explored through online surveys completed pre- and post-implementation of intensive therapy and mapped to domains of the Theoretical Domains Framework (TDF). Translation and behaviour change success will be assessed using the Reach Effectiveness-Adoption Implementation Maintenance (RE-AIM) framework and a combination of qualitative and quantitative data collection methods. These data will assist with the development of an Implementation Toolkit aiding future translation into practice.

**Discussion:**

The embedded process evaluation will help understand the interplay between the implementation context and the intensive therapy intervention following surgery for low-trauma hip fracture. Understanding these mechanisms, if effective, will assist with transferability into other contexts and wider translation into practice.

**Trial registration:**

ACTRN 12622001442796.

**Supplementary Information:**

The online version contains supplementary material available at 10.1186/s13063-024-08143-4.

## Background

Hip fractures are common, increasing in incidence, and have a high burden for patients and the health system. Globally, the incidence of low-trauma hip fractures—typically seen in people with osteoporosis—is anticipated to increase from 1.6 million in 2000 to 4.5 million by 2050 [1]. A large component of the increased burden can be attributed to prolonged hospital length of stay [2]. With an ageing population and increasingly complex care needs, additional research is needed to optimise patient care and improve patient outcomes following hip fracture surgery.

Current evidence demonstrates poor functional outcomes following hip fracture despite prolonged inpatient rehabilitation and significant cost to the healthcare system. In some jurisdictions, the average total length of stay exceeds 30 days [2], and only 24% of patients regain their pre-fracture mobility at 120 days post-fracture [3, 4]. There are significant ongoing costs as a result of hip fractures for the patient, their family and the healthcare system [5].

There is some evidence to suggest that early and intensive rehabilitation in the acute setting could provide opportunities to improve recovery and reduce hospital length of stay following hip fracture [6]. However, current care guidelines for the management of patients following hip fracture do not specify intensity of physiotherapy, reflecting a lack of robust evidence to guide practice [7, 8]. The current HIP fracture Supplemental Therapy to Enhance Recovery (HIPSTER) trial aims to address this evidence gap by conducting a multi-site, double-blinded, randomised controlled trial (RCT) comparing usual care to intensive in-hospital physiotherapy [9]. The HIPSTER trial will follow Medical Research Council (MRC) recommendations for developing and evaluating complex interventions [10]. It will include a process evaluation embedded within the trial with the aim of facilitating research translation within the clinical setting.

The HIPSTER Trial is an Australian Medical Research Future Fund (MRFF) supported trial. It is a two-arm, assessor-blinded randomised controlled trial. A detailed protocol has been published [9] with an overview of key trial design elements presented here. A sample of 620 participants will be recruited from 8 acute hospitals in Australia. To be eligible for inclusion, participants must be aged ≥ 65 years old, be able to mobilise pre-operatively, admitted from home with an isolated subcapital or intertrochanteric hip fracture, suitable for surgical management (including fixation or arthroplasty). For patients unable to provide informed consent due cognitive impairments, consent will be requested from the person responsible for their medical decisions. Consenting participants will be randomised 1:1 to either usual care (physiotherapy as usually provided by that site) or intensive physiotherapy in hospital over the first 7 days following surgery (two additional sessions per day, one delivered by a physiotherapist and the other by an allied health assistant). The aim of intensive physiotherapy is to progress the functional gains achieved in the usual care session as documented in the participant’s medical record. The allied health assistant will aim to practice exercises and functional mobility from the usual care physiotherapy session. A 7-day period was selected as this is the median length of stay in the acute care setting in Australian hospitals [3]. The primary outcome of the HIPSTER trial is total hospital length of stay. Secondary outcomes include clinical outcomes and healthcare costs in the 12 months following hip fracture. The sample size calculations allow for 10% attrition and are based on the primary outcome measure and the results from the phase II trial [6]. Alfred Health Human Research Ethics Committee has approved the protocol for the main trial and this process evaluation (Project ID 8840, local reference 441–22).

### The process evaluation

Implementing intensive physiotherapy following hip fracture requires a system change in usual practice for physiotherapists, allied health assistants and patients. It also requires workforce and other resourcing adjustments. Therefore, it is important to understand barriers and facilitators to implementation of intensive therapy following hip fracture from multiple perspectives. The complexities of the intervention and setting lend itself to completing a theory-based process evaluation in parallel with the HIPSTER clinical trial [10]. This will help identify how the intervention interacts with contextual factors [11] and how these vary across each of the eight sites. A recent update by the MRC identified understanding the implementation context as an important goal in establishing intervention effectiveness [11]. Research into intensive therapy for other conditions (such as stroke) provide insight into what implementation barriers may exist following hip fracture surgery. The A Very Early Rehabilitation Trial after stroke (AVERT) identified key implementation barriers such as staffing, team challenges and organisational barriers [12]. Patient barriers unique to the acute hospital setting included medical stability, severity of condition and participation in therapy [12]. Completing a theory-based process evaluation will expand our understanding of how and why intensive physiotherapy is effective or not effective and therefore aid translation into management of patients following hip fracture surgery [13].

The implementation context and problem inform the following assumptions about effective implementation of the intervention (see Fig. 2). Firstly, intensive physiotherapy improves patient functional outcomes, and this improvement will result in a shorter length of stay. Secondly, it is assumed the staff delivering the intensive therapy have the necessary skills and knowledge to effectively deliver the additional sessions, and it will be feasible to deliver the intensive therapy for at least 4 days in the acute hospital.

### Aims

The aim of the process evaluation embedded within the HIPSTER trial is to support the interpretation of trial outcomes and inform future scale up and facilitate delivery in clinical practice. The process evaluation will answer the following questions:

1. To what extent was intensive physiotherapy delivered to intervention group participants in the first 7 days following hip fracture?
2. What factors are associated with effective implementation of intensive physiotherapy following hip fracture?
3. What barriers and facilitators exist for implementation of the intervention, from the perspectives of healthcare professionals, and the patients who were offered the intervention?
4. How likely is intensive physiotherapy following hip fracture surgery to be sustainable and what factors will enable sustainable translation into clinical practice within the acute hospital setting?

## Methods

**Fig. 1:**
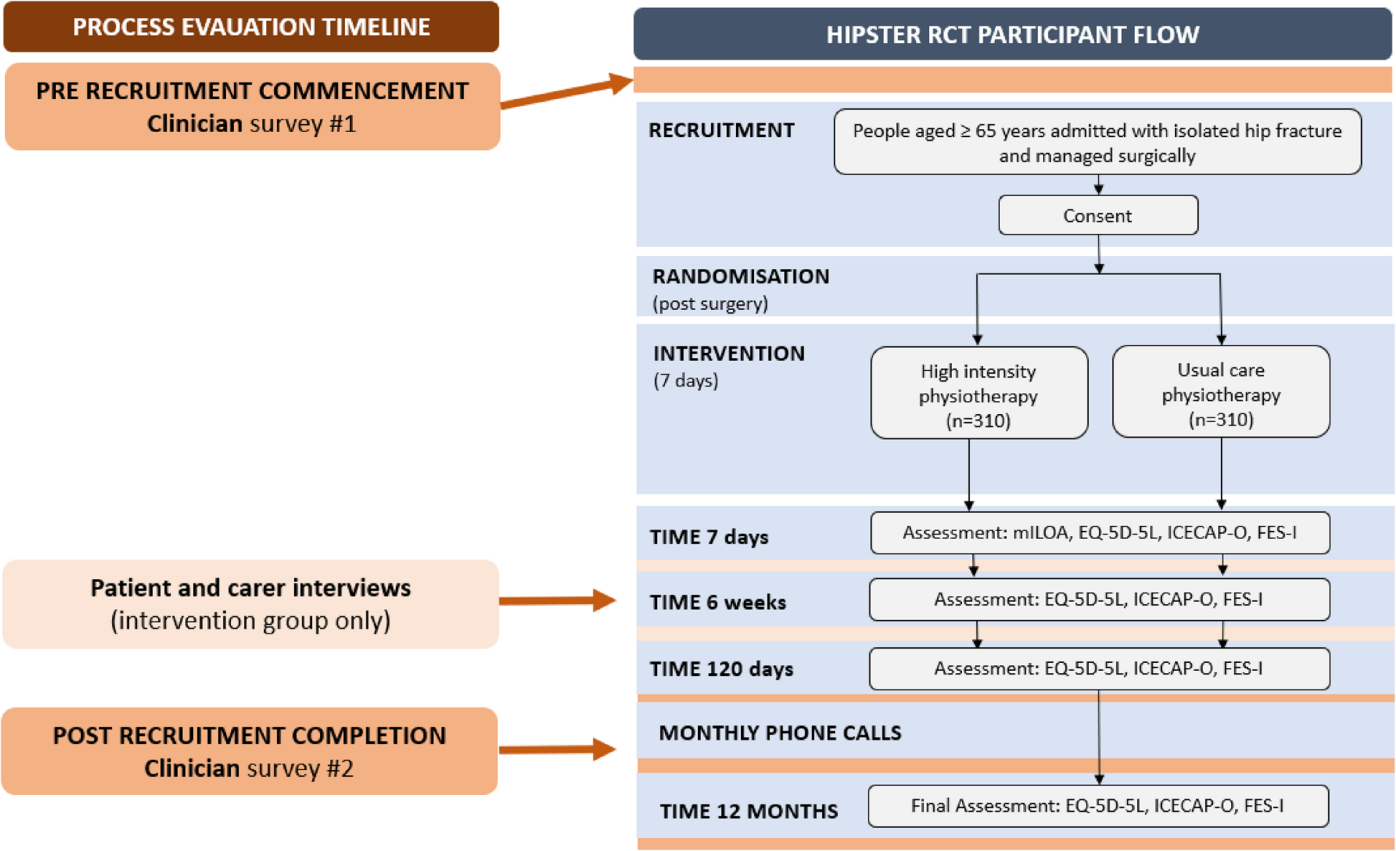
Process evaluation design and conduct. mILOA, Modified Iowa Level of Assistance Score; EQ-5D-5L, EuroQol 5D-5L; ICECAP-O, ICEpop CAPability measure for Older people; short FES-I, Short Falls Efficacy Scale—International

The MRC recommendations used to conceptualise the complex intervention components includes applying a theory-based approach to guide the process evaluation. We will apply two theoretical frameworks: the Theoretical Domains Framework (TDF) [14] and Reach Effectiveness, Adoption, Implementation and Maintenance (RE-AIM) [15], to analyse results and synthesise findings from qualitative and quantitative data collection methods. The study design and timepoints of measurement are in Fig. 1 and Table 1.

**Table 1:** Schedule of trial follow-up and procedures

Assessment/procedure	Baseline	Day 7	Hospital discharge	6 weeks	120 days	12 months
Informed consent	X					
Demographic information and baseline outcomes	X					
Total hospital length of stay			X			
mILOA		X				
EQ-5D-5L and ICECAP-O		X		X	X	X
FES-I		X		X	X	X
Remain in hospital				X		
Return to preadmission mobility				X	X	X
Current residence				X	X	X
Reoperation rate				X	X	X
Discharge destination from the acute ward			X			
Adverse events (including readmissions, falls)				X	X	X
Healthcare utilisation				X	X	X
Economic evaluation						X
Implementation						X

*mILOA* Modified Iowa Level of Assistance Score, *EQ-5D-5L* EuroQol 5D-5L, *ICECAP-O* ICEpop CAPability measure for Older people, *short FES-I* Short Falls Efficacy Scale—International, *MBS/PBS* Medicare Benefits Schedule and Pharmaceutical Benefits Scheme

To determine translation and behaviour change success of HIPSTER, the RE-AIM framework will be applied. This framework effectively evaluates public health interventions by assessing outcomes against five key dimensions: Reach, Effectiveness, Adoption, Implementation and Maintenance [16]. We will report on all domains across individual and organisational levels, where appropriate [15, 16]. The outcomes assessed (Table 2) include participant characteristics, participation rates, intervention completion, intervention effectiveness and characteristics of staff delivering the intensive physiotherapy. Key outcomes will be reported and analysed according to the clinical protocol [10]. Categorical data will be summarised in counts and percentages, with numerical data summarised as either means and standard deviations or medians and interquartile range depending on distribution of data. Qualitative data will be analysed using thematic analysis. The TDF will be applied to understand individual behaviour change success. The TDF is a framework combining different theories of behaviour change to identify factors affecting healthcare professional behaviour and guide more targeted implementation interventions [17]. It will be used as a coding framework to understand clinician responses to the pre- and post-implementation surveys [14, 18].

**Table 2:** RE-AIM elements and outcomes assessed

RE-AIM element	Outcomes assessed
Reach	Percent of eligible individuals who participate Characteristics of participants and non-participants (age, insurance status, postcode, pre-fracture residence, walking ability and cognitive status)
Effectiveness	Clinical outcomes, healthcare utilisation, adverse events Patient and carer experience (semi-structured interviews)
Adoption	Characteristics of staff delivering intervention, health care professional experience (surveys), clinical partner involvement
Implementation	Number of intensive physiotherapy sessions delivered, components delivered, adaptations made to intervention, total physiotherapy time
Maintenance	Individual: patient outcomes at 12 months (e.g. quality of life), factors supporting the participant’s recovery Organisation: intent to continue the intervention at 6 months post-trial completion, local funding models, modifications made, intervention intensity provided to patients

*RE-AIM* Reach, Adoption, Implementation and Maintenance

### Process evaluation design and conduct

A mixed methods study design will be undertaken in parallel to the HIPSTER trial to evaluate the process of HIPSTER at the intervention level, patient and carer level and clinician level.

#### Intervention level

To determine translation at an intervention level, the RE-AIM framework will be applied. A site initiation visit will be completed, providing education about current evidence, existing gaps in the literature and aims of the HIPSTER Trial. No additional training will be provided for staff delivering the intervention. The intervention therapy delivered will be determined by the physiotherapist’s assessment of the individual patient. The physiotherapy review will aim to progress functional gains achieved in the usual care session including increasing independence with mobility, gait aid progression and increasing walking distance. The allied health assistant review will practice achievements gained during the usual care review. The key difference in management between usual care and the intervention group is the increased frequency of physiotherapy and allied health assistant sessions. There should be no change to usual care with all discharge and follow-up determined by usual practices at the site. We will assess any changes to usual care over the course of the trial, including changes to clinician behaviours. Sites will maintain an intervention log for each participant randomised to the intervention group. Staff delivering the intervention will complete it at the end of each session. The intervention log will contain the number of intervention sessions delivered per resource (Physiotherapist or Allied Health Assistant), the duration of the session (minutes) and a brief description of the intervention provided (e.g. ambulation, exercises). Any missed intervention sessions will be documented, and an explanation provided. These data will track intervention fidelity and be summarised by percentage of planned sessions delivered. Intervention completion will be defined as those that received their designated therapy for at least 4 days in the acute hospital.

The key components of the intervention assessed by RE-AIM are outlined in Table 2. The *reach* of the intervention will be determined by the percentage of eligible participants who participate and their characteristics. Intervention *effectivenes*s will be evaluated by clinical outcomes (Table 1 and Fig. 1) as explained in our trial protocol [9], adverse events and patient experience (semi-structured interviews with questions focussing on the perceived effect of the intervention). The primary clinical outcome is total hospital length of stay (days). Secondary clinical outcomes include functional mobility assessed by the Modified Iowa Level of Assistance Score (mILOA) and completed on day 7 following surgery; health-related quality of life and falls efficacy will be assessed at all follow-up timepoints and measured through EuroQol 5D-5L (EQ-5D-5L), ICEpop CAPability measure for Older people (ICECAP-O) and Short Falls Efficacy Scale—International (FES-I). Healthcare utilisation and the associated costs will be collected through monthly phone calls with the participant, and the collection of health systems costs from Health Services Australia Data (incorporating Medical Benefits Schedule and Pharmaceutical Benefits Scheme) over the 12-month follow-up period. Adverse events as defined by GCP standards will include hip and non-hip related in-hospital and out-of-hospital events. The *Adoption* of the intervention including health care professional characteristics, experience and perspectives of intensive therapy will be gathered through pre- and post-clinician surveys. We will record intervention completion, the number and components of the intervention delivered including total physiotherapy and allied health assistant time and adaptations made for *implementation*. *Maintenance* at an individual level will be measured by patient outcomes across at 12 months following surgery (e.g. quality of life measured using EQ-5D-5L) as well as the participant and carer experience of the intervention and the factors supporting their recovery. *Maintenance* at an organisational level will be measured by intention to continue delivering the intervention at 6 months following trial completion, including local funding models and modifications made, and the intensity of the intervention delivered.

#### Participant and carer level

Clinical outcomes and their collection method are explained in detail in our published protocol [9]. Eligible patients will be approached to consent to the trial during the perioperative phase. They will be included if they fulfil the following criteria: age $\ge$ 65 years old, admitted from home with a hip fracture (either subcapital or intertrochanteric) and managed surgically. They will be excluded if they were unable to mobilise (with or without a gait aid prior to the surgery) and are not allowed to full-weight-bear or weight-bear-as-tolerated. Once consented, baseline data will be collected at hospital admission with key follow-up assessment timepoints occurring at day 7, 6 weeks, 120 days and 12 months following surgery. At initial study consent, participants or their proxy decision-maker will be invited to participate in semi-structured interviews at 6 weeks following surgery. At the 6-week review with the blinded assessor, participants or their carers will be asked if they consent to be contacted by an experienced qualitative researcher who is independent of the site clinical and research staff. Those who consent and are eligible (have recall of the acute inpatient hospital admission) will be contacted to participate in a semi-structured interview. The participants will be asked about their experience of intensive therapy and their recovery and discharge from hospital as well as barriers and facilitators to participation in the intervention, with an interview guideline developed using RE-AIM framework (supplementary material A). Interviews will be conducted via the telephone, at a time of the participant’s preference. A sampling framework will be applied to ensure diversity across hospital, gender and presence of a carer. Interviews will be transcribed verbatim within 6 weeks of completion of the interview. All participant discussions and responses will be coded to ensure participants cannot be identified from their responses. Two researchers will independently undertake line by line analysis of the coded transcripts, to generate descriptive themes [19]. The two researchers will refine the descriptive codes into themes and subthemes through iterative discussion [20]. Representative quotes from participants will be used to illustrate the identified themes. Interviews will continue until data saturation is reached, i.e. when no new themes emerge from consecutive interviews.

#### Clinician level

Pre-implementation and post-implementation clinician surveys linked to domains of the TDF will explore behaviours that act as barriers and facilitators to implementation of HIPSTER. Any clinician involved in the care of patients following hip fracture will be invited via email to anonymously participate in an online survey (Qualtrics®, UT, USA) by their site principal investigator prior to the commencement of recruitment. The pre-implementation online survey will comprise 25 questions each relating to the domains of the TDF (Table 3) as well as an additional five questions relating to the study protocol. Participants will be asked to rate level of agreement to a statement using a six-level Likert scale ranging from ‘completely agree’ to ‘completely disagree’. These same questions will be repeated in the post-implementation survey. Free text options will also allow clinicians to list any perceived barriers and facilitators to implementation of intensive therapy following hip fracture. The post-implementation survey will be distributed 7 days following completion of participant recruitment to prevent unblinding staff. The survey will include an additional 28 statements and short answer questions relating to barriers and facilitators to implementation, suitability of the site and trial design and likelihood to continue providing high-intensity therapy following hip fracture. In both surveys, we will collect demographic information about the clinician, their qualifications, years of experience and professional delegation. We will aim to collect at least the same number of pre-implementation and post-implementation surveys from each of sites.

**Table 3:** Survey evaluation of clinician perspective on providing intensive physiotherapy following hip fracture surgery

TDF domain	Number of questions	Representative question
Knowledge	**3**	I am aware of the findings of the High Intensity Physiotherapy following Hip fracture trial (H4H) published in the MJA in 2016
Skills	**2**	I have the ability to deliver high-intensity physiotherapy for hip fracture patients (usual care plus two additional daily sessions, one delivered by a physiotherapist and one delivered by an allied health assistant)
Social/professional role	**2**	There has been sufficient local clinician time allocated for training to deliver high-intensity therapy for hip fracture patients
Beliefs about capabilities	**4**	I feel confident to discuss the pros and cons of high-intensity physiotherapy post hip fracture surgery with patients
Optimism	**2**	I don’t think that all patients are capable of participating in high-intensity physiotherapy post hip fracture surgery
Beliefs about consequences	**4**	Whether a patient receives usual care or high-intensity physiotherapy after hip fracture doesn’t really matter
Reinforcement	**2**	Whenever I look for ways to improve care for patients who have had a hip fracture, I get recognition from professionals who are important to me
Intentions	**3**	I intend to support other staff to develop skills to deliver high-intensity physiotherapy post hip fracture
Goals	**1**	I believe that offering inpatient rehabilitation to patients following hip fracture surgery is important
Memory, attention, decision processes	**1**	I am familiar with the mobilisation guidelines for post-operative mobilisation following hip fracture (Australia New Zealand Hip Fracture Registry or NICE Guidelines)
Environmental context and resources	**5**	Our institution offers mobilisation on the first post-operative day to all patients following hip fracture surgery
Social influences	**4**	Most people whose opinion I value would support offering high-intensity physiotherapy to our patients
Emotion	**2**	I feel nervous about sending patients directly home (rather than to inpatient rehabilitation) after a hip fracture
Behavioural regulation	**1**	I am confident I could provide high-intensity physiotherapy for patients following a hip fracture

*TDF* Theoretical Domains Framework

Survey responses will be reported descriptively with the number and proportion of respondents. Themes emerging from free text responses will be mapped to the TDF components [14]. The comparison of pre- and post-implementation survey responses will enable differentiation between perceived and experienced barriers and facilitators, which will be used to guide future implementation. These clinician experiences will help with the creation of interventions and an implementation toolkit to target key behaviours of change with the goal of facilitating sustainable implementation.

#### Logic model

**Fig. 2:**
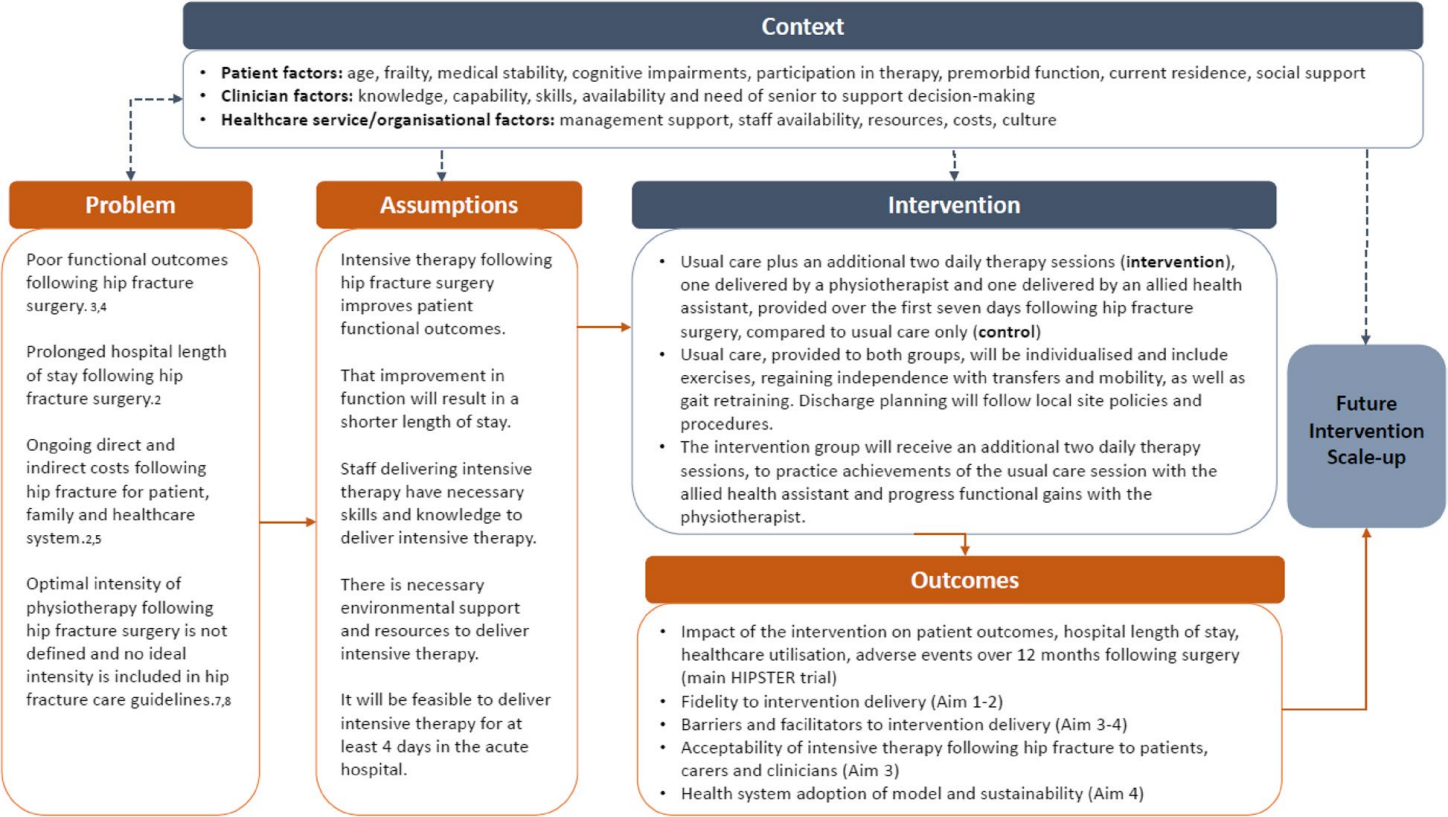
Logic model for the process evaluation of HIPSTER

As per best-practice methods for evaluation of complex interventions [10], a logic model was created outlining key factors that may influence implementation, proposed causal mechanisms and target outcomes (Fig. 2).

## Discussion

This study will be the first process evaluation of intensive in-hospital physiotherapy delivered in the acute setting for patients following surgery for low-trauma hip fracture, conducted alongside a randomised controlled trial. The process evaluation applies evidence-based theoretical frameworks, TDF and RE-AIM, and follows MRC recommendations [10]. This creates a robust study design to address the study aims by comprehensively evaluating the contextual factors, patient and clinician factors that may contribute to intervention effectiveness.

We recognise some limitations of the proposed approach. It is anticipated that clinician turnover and staffing changes may impact the number of clinicians that complete both pre- and post-implementation surveys, making direct comparisons difficult to ascertain. Furthermore, due to resourcing limitations, the patient and carer interviews will be limited to English-speaking participants, potentially resulting in underrepresentation of the experience of people from culturally and linguistically diverse groups. While a detailed logic model has been created aligning with key MRC recommendations, there may be unmeasured pathways or mechanisms of effect that cannot be feasibly explored.

If the intensive in-hospital physiotherapy following hip fracture (HIPSTER) is found to not be effective, the process evaluation will provide critical information about the contributing contextual factors. Such findings will have implications on future directions of research within this population. Conversely, if HIPSTER is effective, the findings from the process evaluation will help guide future scale up and wider translation into other settings [13]. An Implementation Toolkit informed by the findings of this process evaluation will be used to facilitate broader adoption and implementation of HIPSTER. The toolkit will be openly available and disseminated through key partners of the study including the Australia and New Zealand Hip Fracture Registry (ANZHFR) with the aim to improve outcomes for more patients following hip fracture surgery. The uptake of the implementation toolkit will be tested more broadly including in international settings.

## Trial status

This manuscript reports protocol version number 4 of 10 March, 2023. Trial recruitment began in January 2023 and is ongoing. Trial recruitment is anticipated to be completed by June 2024.

### Supplementary Information

Supplementary Material 1.

## Data Availability

The coordinating principal investigator (Dr Lara Kimmel) will have access to the final trial dataset. Any requests for data to support the protocol can be made to the principal investigator (Dr Lara Kimmel).

## Abbreviations

MRC: Medical Research Council
RE-AIM: Reach, Effectiveness, Adoption, Implementation, Maintenance (framework)
TDF: Theoretical Domains Framework
