# Supplementary material for: Implementation of supplemental physiotherapy following hip fracture surgery: a protocol for the process evaluation of a randomised controlled trial
Source: Trials. 2024 May 24;25:344. doi: 10.1186/s13063-024-08143-4 (PMC11127386; doi:10.1186/s13063-024-08143-4)
Supplement: Supplementary file 1 — Supplementary Material 1. [file 13063_2024_8143_MOESM1_ESM.docx]

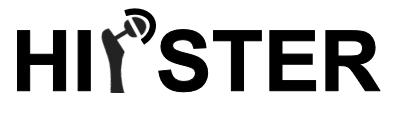


**PARTICIPANT**

**DISCUSSION GUIDE**

Version 1, July 22 2022

HIPSTER: Hip fracture Supplemental Therapy to Enhance Recovery

1. **Facilitator’s Welcome and Consent Statement prior to audio-taping.**
2. **Interview guide for participant interviews (people who have had a hip fracture and consented to participate in the HIPSTER trial)**

**Facilitator Welcome & Consent Statement**

**Process Evaluation of the HIPSTER Trial**

***Facilitator’s welcome, introduction and instructions to participants: This is to be read aloud prior to all interviews.***

**Welcome** and thank you for volunteering to take part in this interview. You have been asked to participate as your point of view is important. I realise you are busy and I appreciate your time.

**Introduction:** We would like to hear your thoughts and feelings about the physiotherapy you completed in the first week after your hip operation. Our discussion will take 30 – 60 minutes, and of course you may stop at any time or not talk about anything we bring up.

If it is OK with you, I would like to tape this interview so that I can later write down what you said without mistakes, this is known as transcribing an interview. Once I have transcribed the interview, I will delete the audio recording. May I record the discussion to ensure we are accurately recording what you have discussed? *[if yes, switch on the recorder].*

*Anonymity:* Despite being recorded, I would like to assure you that the discussion will be anonymous- this means that it will not be possible to know who said what, and not even which hospital they have come from.

The recording will be kept as an electronic file until it is transcribed word for word.

The transcribed notes of this guided discussion will contain no information that would allow you to be identified.

*Voluntary Participation:* We also need to make sure you know that if there are any questions or discussions that you do not wish to answer or participate in, you do not have to do so. I want to hear your story and your experience but if at any time you don’t want to answer one of my questions, or you don’t want to continue talking about something, please tell me and we will move on and not go back to it. And, we can also stop at any time if you wish.

*Consent:* Before we begin, I do need to ensure that I have checked that you still consent to us talking today, and also to us recording this discussion. (Do you mind saying yes out aloud please). Thankyou.

| **HIPSTER Trial Process Evaluation discussion guide** |
| --- |

**Purpose of the interview**: Understand the person’s experience of intensive physiotherapy. Factors which contributed to their outcomes and participation. What were their expectations and were they met? What were the perceived impacts of intensive physiotherapy on their treatment goals and outcomes?

*The interview will focus on the initial days (i.e. up to day 7) following the participant’s surgery for their hip fracture.*

| **Area of Interest** | **Initial Broad Descriptive Questions** | **Possible Probing Questions**  (*These are a guide only. Depending on what the patient tells you, you do not have to ask all these questions or use the words exactly as written.)* |
| --- | --- | --- |
| **Reach** | Tell me about your experience with physiotherapy following your hip surgery? | - How did you feel when the physiotherapist or allied health assistant arrived? |
| **Effectiveness** | Did you feel like the physiotherapy provided met your needs? | - If yes, how did it meet your needs? - If no, what was missing? |
| **Adoption** | Can you describe the physiotherapy you received at the hospital in the first few days following your hip fracture? | - What did you like? - What did you dislike? |
| **Implementation** | What did you think about the amount of therapy (PT and AHA) you received? | - Would you want more or less therapy? - How would you feel if you had more therapy? |
| **Maintenance** | What do you feel helped your recovery following your hip fracture?  Can you tell us about your discharge from hospital?  Can you tell me about any part of your care that helped to prepare you for going home or discharge from hospital? | - Did you go home or to another hospital after your care? - Did you feel ready to go home when you did and what could have helped you feel more comfortable? |
| **Concluding**  **Questions** | Is there something else that you would like to say, that we have not talked about in this interview? | |
